# Dietary Polyphenols, Resveratrol and Pterostilbene Exhibit Antitumor Activity on an HPV E6-Positive Cervical Cancer Model: An *in vitro* and *in vivo* Analysis

**DOI:** 10.3389/fonc.2019.00352

**Published:** 2019-05-09

**Authors:** Kaushiki Chatterjee, Sumit Mukherjee, Jonathan Vanmanen, Probal Banerjee, Jimmie E. Fata

**Affiliations:** ^1^Doctoral Program in Biology, CUNY Graduate Center, New York, NY, United States; ^2^Department of Biology, College of Staten Island, New York, NY, United States; ^3^Doctoral Program in Biochemistry, CUNY Graduate Center, New York, NY, United States; ^4^Department of Chemistry & The Center for Developmental Neuroscience, City University of New York at The College of Staten Island, New York, NY, United States

**Keywords:** HPV E6 positive cervical cancer, natural product, resveratrol, pterostilbene, PCNA, caspase-3, VEGF, *in vivo*

## Abstract

Human papilloma virus (HPV)-induced cervical cancer is one of the most frequent cancers in women residing in underdeveloped countries. Natural compounds like polyphenols continue to be of scientific interest as non-toxic effective alternative treatments. Our previous work showed the efficacy of two polyphenols, resveratrol, and pterostilbene on human HeLa cells. Here we explored the *in vitro* anti-cancer activity and *in vivo* anti-tumor potential of these two structurally similar compounds on HPV oncogene E6 and E7 positive murine TC1 cells. *In vitro* analysis confirmed the cytotoxic potential of both resveratrol and pterostilbene compounds with each having a low IC_50_ value and each showing the ability to downregulate viral oncogene E6. Further *in vivo* studies on TC1 tumors developing in mice indicated that treatment with either resveratrol or pterostilbene can significantly inhibit tumor development, with both compounds capable of downregulating E6 and VEGF tumor protein levels. Interestingly, the decrease in tumor size in pterostilbene was associated with tumor cell apoptosis, as indicated by an upregulation of activated caspase-3 whereas in resveratrol-treated mice it was accompanied by arrest of cell cycle, as indicated by a downregulation of PCNA. Thus, resveratrol and pterostilbene can serve as potential antineoplastic agents against HPV E6+ tumors and may suppress tumor growth via two different mechanisms.

## Introduction

Human papilloma virus (HPV) is responsible for almost all cervical cancer incidences (1). High risk HPV types are also responsible for other types of invasive malignancies including head and neck cancers, oropharyngeal cancers, and other anogenital cancers. Cervical cancer is the fourth most common cancer in women and accounts for almost 7.5% of female cancer deaths worldwide (2). With current screening and vaccination programs, the rate of cervical cancer has decreased in developed countries, however the major burden lies within developing countries due to lack of awareness and resources (3, 4). Every year more than 500,000 new cases of cervical cancer are reported, with the majority originating from developing countries. Etiologically, prolonged infection with HPV has been the primary agent associated with cancerous progression. Moreover, HPV16 and HPV18 are the two most prevalent high-risk strains that are responsible for most of the cervical cancers diagnosed. Sustained expression of two oncoproteins, E6 and E7, play an essential role in the maintenance of these malignancies (5). All of the current treatment regimens for metastatic cervical cancer (radiation, surgery, and chemotherapy) (6) are associated with deleterious side effects (7–9). Advancements in cancer prevention and treatment have unfortunately failed in significantly decreasing cervical cancer mortality in developing countries, often because of the high costs associated with each. Due to a growing need for effective low-cost cervical cancer therapy with no detrimental side effects, naturally occurring dietary polyphenols, that have known anti-cancer properties, have continued to be of great interest.

Stilbenes are a group of naturally occurring polyphenols that have a diphenylethylene nucleus and are synthesized in plants as a defense mechanism (10). Resveratrol, commonly found in specific fruits including grapes and mulberries, is the most well-studied stilbene compound with considerable evidence supporting its anti-cancer properties (11). Another such compound with a stilbene core that has gained much attention in the recent years is pterostilbene, a structural analog of resveratrol. Several studies indicate that pterostilbene has a higher bioavailability than resveratrol and like resveratrol is nontoxic to humans even at high doses (12, 13).Studies using pterostilbene on normal liver cells and non-cancerous breast epithelial cells show specificity against cancerous cells without causing any acute toxicity to normal cells (14, 15).Clinical trials in humans have shown that both these compounds are nontoxic to normal cells and are well-tolerated (13, 16). Both resveratrol and pterostilbene have shown much potential in inhibition of different types of cancer cells including colon cancer, stomach cancer prostate cancer, and breast cancer (17, 18). Few studies have utilized *in vivo* tumor models to determine the effectiveness of these two compounds and these involved pancreatic, colon, and liver cancer (11, 19–23). Previous studies by our team using these two compounds have shown that both resveratrol and pterostilbene are cytotoxic to HPV18 HeLa cells in a dose dependent manner (4). The compounds were able to inhibit cell cycle progression, decrease migration at lower concentrations and also induce apoptotic cell death at higher concentrations *in vitro* (4). Like our previous finding, other studies also indicate that pterostilbene might have superior efficacy in combating cancer (4, 24). We were, to the best of our knowledge, the first group to report that these polyphenols were able to specifically target the essential oncoprotein E6 and induce cervical cancer cell death (4).

In the current study we set out to examine the efficacy of both these compounds *in vivo*. For this purpose, we used a cervical cancer murine model injected with TC1 cells. These cells were originally derived from mouse primary lung epithelial cells transformed with three oncogenes: HPV16-E6, -E7, and c-Ha-Ras. TC1 tumor models have been widely used as a model for cervical cancer and have been used to study *in vivo* effects of several therapeutic strategies (25–27). Unlike resveratrol there are very few studies on the effects of pterostilbene in cervical cancer. Furthermore, there is very little known about the anticancer effects of resveratrol and pterostilbene when compared *in vivo*. In this study comprehensively analyzed the efficacy of these two polyphenols in HPV-oncogene positive tumors and explored the therapeutic possibility of these natural compounds *in vitro* and *in vivo*. Our findings show for the first time that these compounds can significantly reduce the size of HPV-oncogene positive TC1 tumors. Importantly, both compounds significantly reduced HPV E6, the oncoprotein driving tumor formation. Only a short treatment cycle of 5 days was sufficient to see a significant antitumor potential of resveratrol and pterostilbene. We propose that this antitumor efficacy is dependent on two different mechanisms where resveratrol playing a vital role in arresting tumor growth evident by a significant reduction of proliferating cell nuclear antigen (PCNA) and pterostilbene largely causing cell death in tumors by activating apoptotic caspase-3. These results show for the first time that stilbene compounds resveratrol and pterostilbene show a robust antitumor potential both *in vitro* and *in vivo* in HPV E6 positive cancers and are promising candidates as natural anticancer therapeutics.

## Materials and Methods

### Cell Culture

TC-1 mouse cells were procured from the lab of Dr. T.C. Wu at Johns Hopkins University. TC-1 cells were derived from primary lung epithelial cells of C57BL/6 mice after co-transformation with HPV16-E6, HPV16-E7, and c-Ha-Ras oncogenes (25). The cells were maintained in Dulbecco's Modified Eagle Medium: Nutrient Mixture F-12 (DMEM/F-12) (HyClone, GE Healthcare Life Sciences, Manassas, VA, USA), supplemented with 10% fetal calf serum (HyClone, GE Healthcare Life Sciences) with 0.1% Penicillin-Streptomycin Solution (HyClone). Cells were cultured in a humidified incubator at 37°C incubator and 5% CO_2_.

### Determination of IC_50_ Using WST-1 Assay

Four thousand TC-1 cells were plated on 96-well plates and cultured for 24 h. Resveratrol (Acros, #430075000) or pterostilbene (TCI, #P1924) were serially diluted from 5 to 100 μM in DMEM/F-12 supplemented with 1 × insulin-transferrin-selenium (ITS; Invitrogen). Treatment was carried out with the dilutions (in triplicate) for 72 h after which time a WST-1 (Water Soluble Tetrazolium salt-1; Clontech, Mountain View, CA, USA) cell viability assay was performed. The medium was aspirated after treatment and rinsed three times with 1 × Phosphate Buffered Saline (PBS). After rinsing, 80 μL of a 10% WST-1 fresh solution was prepared in DMEM and subsequently added to each well. The plate was then incubated for 1 h at 37°C to get a color reaction. The absorbance was recorded at 440 nm using a 96-well spectrophotometer. The results were then analyzed using GraphPad Prism 7 software to obtain the IC_50_ using a standardized method (4).

### Immunocytochemistry

TC-1 cells were cultured on 8-well chamber slides for 48 h prior to immunocytochemistry. The cells were then untreated (control) or treated with resveratrol (30 μM) or pterostilbene (30 μM) for 48 h prior to immunocytochemical detection of E6. The dilutions of resveratrol and pterostilbene were done in serum-free DMEM/F-12 containing 1% supplement (ITS; insulin, transferrin, selenium; Gibco BRL, Grand Island, NY, USA). After treatment, cells were fixed in 4% paraformaldehyde at room temperature. After rinsing with 1 × PBS cells were permeabilized and blocked with 10% goat serum, 2% bovine serum albumin, and 0.5% Triton X-100 in PBS for 1 h. This was followed by overnight incubation with E6 antibody (sc-460; Santa Cruz Biotechnology) in blocking buffer. After probing with primary antibody, the cells were washed and incubated with Fluorescein isothiocyanate (FITC) conjugated secondary antibodies for 3 h, followed by incubation with 4′,6-diamidino-2-phenylindole (DAPI) (10 μg/mL). After washing with 1X PBS the slides were mounted with coverslips and cell images were acquired using a Leica SP2 confocal microscope. Images of randomly chosen field from different treatment wells were taken and used for quantification. ImageJ was used to measure the fluorescence intensity and cell counting. The fluorescence intensity of E6 antibody was normalized to DAPI intensity (blue).

### TC-1 Mouse Model Generation and Treatment

Fifty thousand TC-1 cells harvested from actively growing cultures were injected subcutaneously in the nape of the neck of female C57BL/6 mice, which were ~2–3 months old. This tumor model has been standardized and used in previous studies (27, 28). Once palpable tumors were formed (in ~15–20 days) treatment cycles was initiated. The mice were anesthetized by intraperitoneal injection of 100 mg/kg ketamine and 10 mg/kg xylazine. A power test was used to determine the minimum number of mice (n) to be used for the study to achieve a confidence level >90% (29). Treatment involved the mice being randomly divided into three groups, control, resveratrol, and pterostilbene–with each group containing 5 mice. Tumors were marked into four quadrants and 10 μl of the 1 mM resveratrol or 1 mM pterostilbene was injected into each quadrant intralesionally for 5 consecutive days. We approximated the average initial tumor volume to be around 0.3–0.5 ml using a caliper to measure approximate tumor diameter (27). We administered 10 μl treatment in 4 quadrants such that the expected internal concentration is above 80 μM (lethal dose for both resveratrol and pterostilbene). The control group of mice were subjected to 1 × PBS injections also for 5 consecutive days. After the completion of the treatment cycle the mice were sacrificed and the tumors were excised. Tumor volumes for the excised tumors were measured using a caliper and volumes were calculated as previously mentioned (27). Tumors were then fixed and processed for immunohistochemistry. All mouse experiments were performed according to NIH guidelines for animal use and approved by the Institutional Animal Care Committee (IACUC) of the College of Staten Island (CUNY) (approval # 11–008).

### Immunohistochemistry

Tumor sections that were taken from the approximate center of the tumor were randomly selected from each group for immunohistochemical analysis. The sections were first subjected to antigen-retrieval using formamide: 2 × SSC at 55°C for 2 h followed by two PBS washes. The sections were then blocked with 3% goat serum or 10% rabbit serum for 2 h and then treated overnight with primary antibodies: anti-E6 antibody (sc-460; Santa Cruz Biotechnology) or VEGF (P-20; Santa Cruz Biotechnology) or PCNA (#2586 Cell Signaling Technology) or Cleaved Caspase-3 (Asp 175) (#9661; Cell Signaling Technology). The antibodies were diluted in 2% goat serum or 2% rabbit serum and 0.1% Triton X in PBS. The sections were washed with PBS after the primary treatment and then probed with the secondary antibody. For E6 primary antibody the secondary antibody used was Alexa Fluor-488 goat anti-mouse (Invitrogen) and for VEGF primary the secondary antibody used was Alexa Fluor-488 rabbit anti-goat (Invitrogen). Sections were also stained only with the secondary antibodies as a negative control for the experimental setup. After overnight incubation with secondary antibody at 4°C the sections were washed and stained with nuclear stain DAPI (10 μg/ml) for 15 min at room temperature. The sections were then mounted on a slide with Gold anti-fade mounting fluid. Confocal Images of the immunostained sections were taken using a Leica SP2 microscope from multiple randomly chosen fields of multiple sections.

### Image Quantification and Data Analysis

All images taken were quantified using ImageJ software. The empirical data obtained from all experiments were analyzed using GraphPad Prism 7 software.

### Statistical Analysis

Statistical analyses were performed using GraphPad Prism®7 (GraphPad Software, Inc., La Jolla, CA, USA). The mean and standard error mean was obtained for each of the groups. One-way ANOVA with *post-hoc* Tukey test was used to compare the data sets and determine the significance as performed in previous publication (4). *p* < 0.05 was considered as significant.

## Results

### Resveratrol and Pterostilbene Are Cytotoxic to TC1 Cells

**Figure 1:**
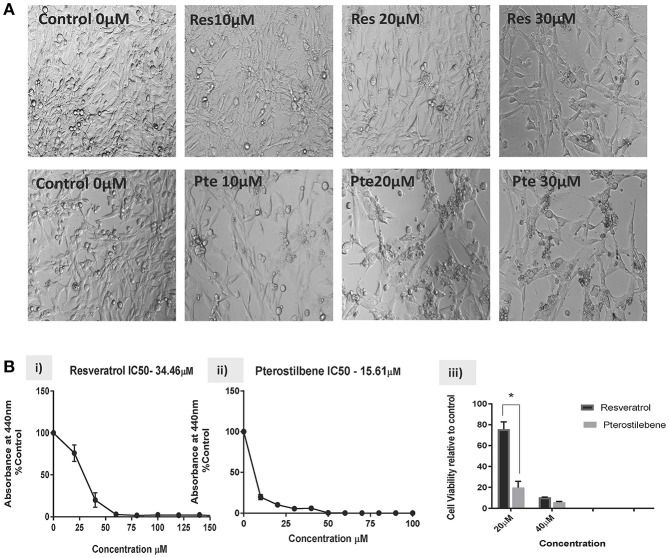
Resveratrol and pterostilbene are cytotoxic to TC1 cells. **(A)** Brightfield analysis of TC1 cells untreated (Control) or treated with 10, 20, and 30 μM of resveratrol (Res) or pterostilbene (Pte). . **(B)** IC_50_ values generated using a water soluble tetrazolium salt-1 (WST-1) assay after 72 h of exposure to resveratrol or pterostilbene shows that pterostilbene **(Bi)** is more cytotoxic than resveratrol **(Bii)** (IC_50_ = 15.61 μM vs. IC_50_ = 34.46 μM; *p* < 0.0001, *n* = 3). **(Biii)** Bar graph represents comparative data showing the difference in viable cells treated with resveratrol or pterostilbene at 20 or 40 μM (mean ± S.E.M.; ^*^p < 0.05, *n* = 3).

Based on the findings of our previous studies on HeLa cells showing the cytotoxic potential of resveratrol and pterostilbene (4), we wanted to determine their anticancer efficacy on HPV-E6-positive mouse TC1 cells. Brightfield images were taken after 48 h of treatment with different concentrations of resveratrol or pterostilbene to see their cytotoxic potential relative to untreated control cells (Figure 1A). A qualitative analysis of the images indicates that compared to control untreated cells, both resveratrol and pterostilbene show significant cytotoxicity, with pterostilbene showing more cell death and cytoplasmic blebbing than resveratrol after 48 h. These comparative effects are visible even at low concentrations of 10 μM and apoptotic cell numbers increased in a dose dependent manner for both treatment groups. Analytically we ascertained the IC_50_ concentrations of these two polyphenols by performing WST-1 assays for cell viability (Figure 1B). The results showed the IC_50_ of resveratrol to be 34.46 μM (Figure 1Bi) while that of pterostilbene was >2-fold less at 15.61 μM (Figure 1Bii). These IC_50_ values were significantly different from each other (*p* < 0.0001) and confirm the superior cytotoxicity of pterostilbene over resveratrol *in vitro* on TC1 cells (Figure 1B).

### E6 Oncoprotein Gets Downregulated by Resveratrol and Pterostilbene *in vitro*

**Figure 2:**
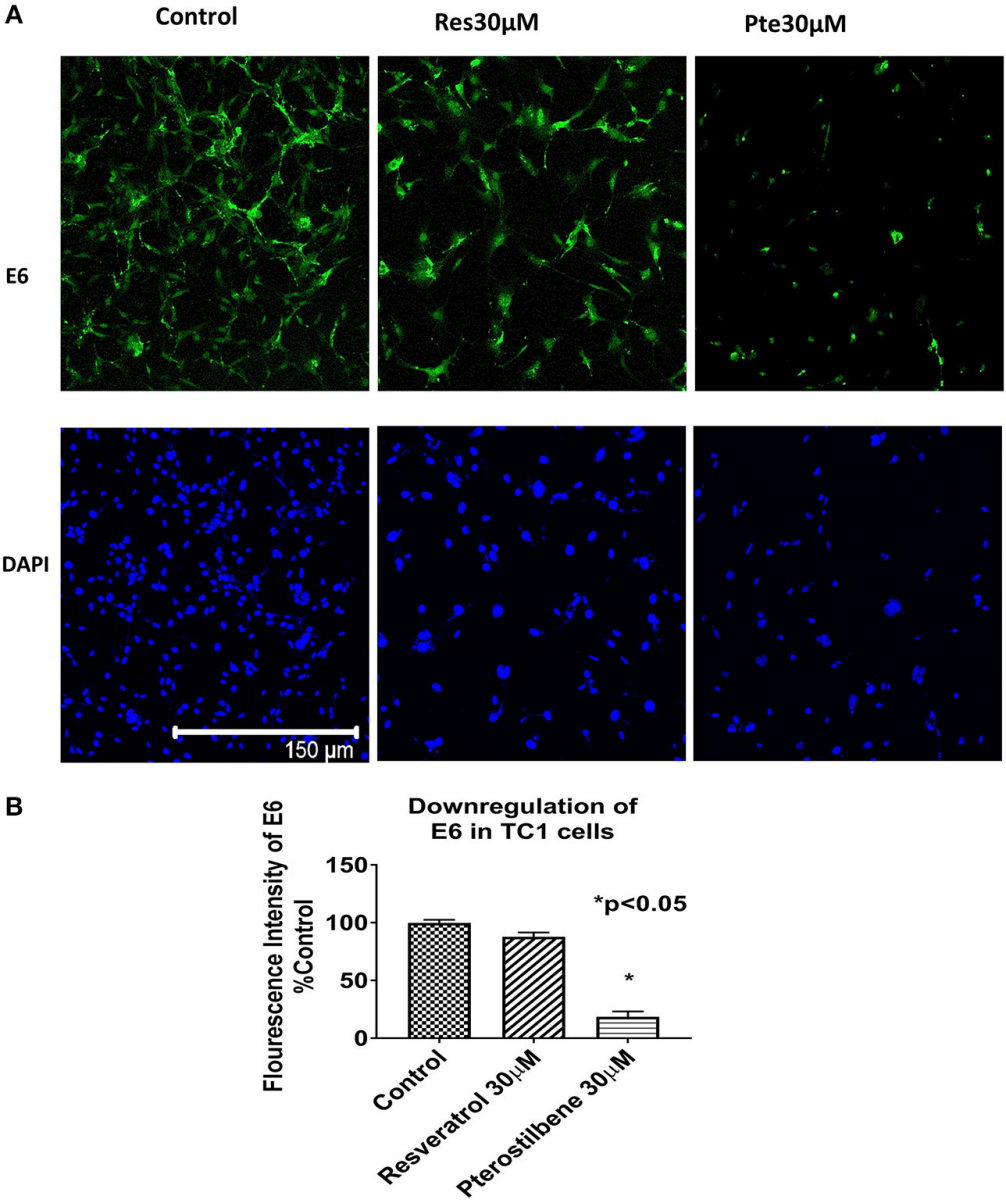
Viral oncoprotein E6 gets downregulated in TC1 cells treated with resveratrol or pterostilbene. **(A)** TC1 cells immunostained and probed for E6 oncoprotein (green) and counterstained with nuclear stain DAPI (blue). Cells treated for 48 h with resveratrol (30 μM) or pterostilbene (30 μM) shows pterostilbene has a more significant downregulation of E6 protein expression as compared to resveratrol and control. **(B)** Bar graph indicates the percent decrease in E6 expression in treated TC1 cells as compared to control untreated TC1 cells. The graphs represent data from three independent experiments (mean ± S.E.M.; ^*^p < 0.05).

To investigate whether the expression of HPV viral oncoprotein E6 was attenuated by either polyphenol treatments, we used immunocytochemical techniques to probe for protein levels in TC1 cells. Using the results from the brightfield images and the IC_50_ values we selected a supra IC_50_ concentration at 48 h for treatments (30 μM). Immunocytochemical analysis indicated that cells treated for 48 h with pterostilbene but not resveratrol had significantly lower E6 levels when compared to control (81 vs. 12%, Figures 2A,B; *p* < 0.0001). Although E6 expression levels in resveratrol treated cells were downregulated by 12% compared to control this down regulation was not statistically significant (Figure 2B; *p* = 0.1838). The downregulation of E6 levels by pterostilbene was also significantly different than resveratrol (*p* < 0.0002). Therefore, our results show that *in vitro* pterostilbene is more potent in downregulating the essential oncoprotein E6 in TC1 cells as compared to resveratrol treatment.

### Reduction of Tumor Size in Mouse Model by Resveratrol and Pterostilbene

**Figure 3:**
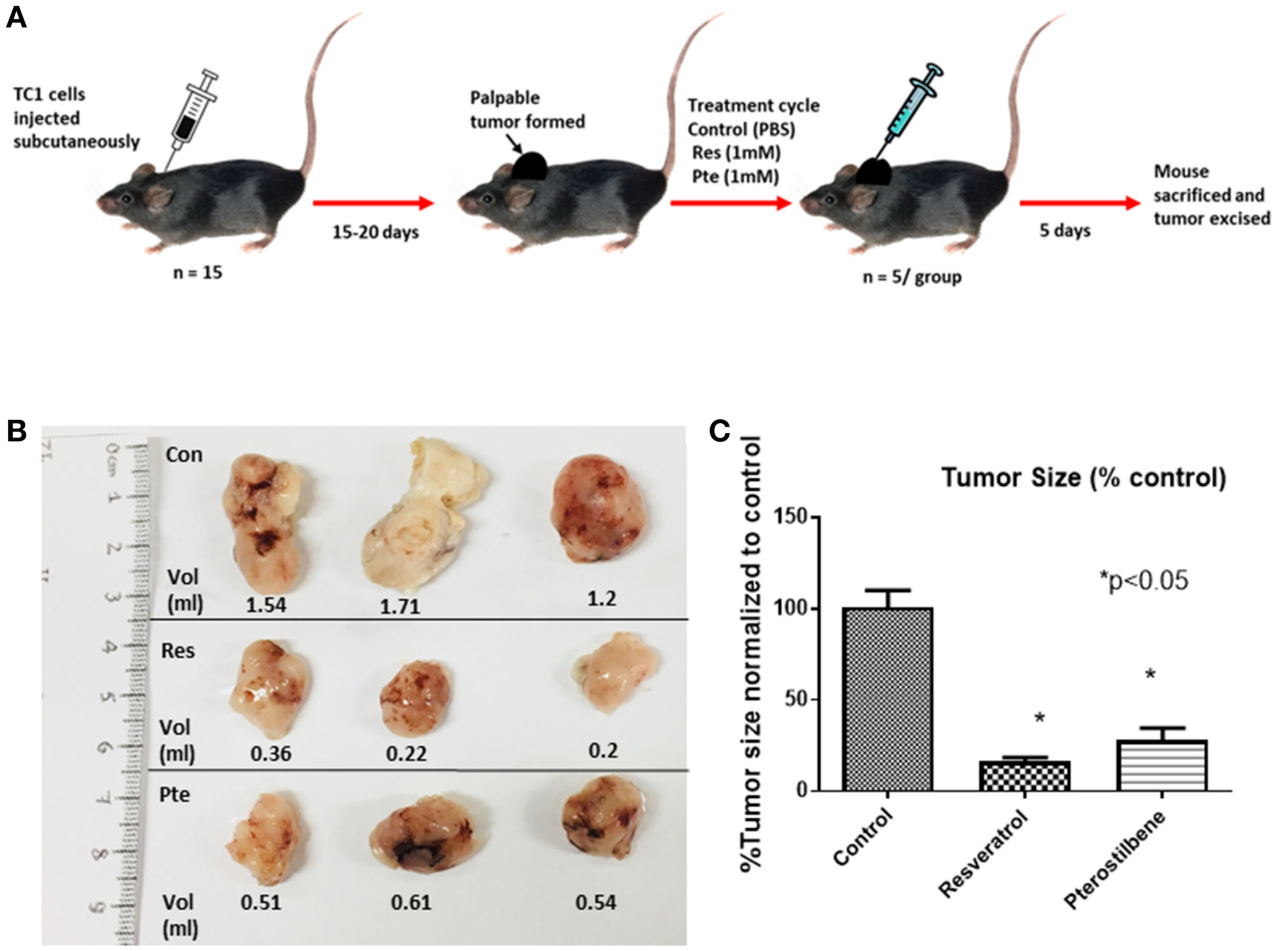
Inhibition of tumor growth in E6+ tumor bearing mouse. **(A)** Schematic representation of mouse TC1 tumor *in vivo* tumor generation and subsequent treatment cycle. TC1 cells were injected subcutaneously in 15 mice followed by daily palpations. After 15–20 days, when the tumors became apparent mice were divided into three groups of five and treated intralesionally with either PBS (control), resveratrol or pterostilbene for 5 consecutive days. After 5 days the mice were sacrificed, and the tumor excised. **(B)** Tumors excised from the three representative groups, displayed for imaging with corresponding volume (ml). In both treatment group tumors show marked decrease in tumor size compared to control. **(C)** Bar graph shows percent reduction in tumor size in mice treated with resveratrol or pterostilbene in comparison to control tumor. Tumor size reduction is significant in the treated groups. The graphs represent data from 5 mice from each group (mean ± S.E.M.; ^*^p < 0.05).

To find out the potency of these two polyphenols *in vivo* we generated mouse tumors by implanting TC1 cells subcutaneously in syngeneic C57Bl/6 female mice. After palpable tumors were formed mice were randomly divided into 3 groups. We treated the mice intralesionally (10 μl in each of the 4 quadrants of individual tumor) with 1 mM resveratrol or 1 mM pterostilbene for 5 consecutive days. Control group mice received PBS injections. The dosage was selected such that the intratumoral concentration of the compounds would be expected to be above lethal dose (calculated from the IC50 curve) in both treatment groups. Treatment was carried out for a period of 5 days to comply with ethical standards and avoid morbidity. After completion of the treatment cycle the tumors were excised, the volume of the tumors were measured using a caliper and then volumes were calculated as mentioned previously (27) (Figure 3A).The tumor size was drastically lower than the untreated tumors for both the treatment groups (Figure 3B). Tumor size is 16.48% (±1.99) in resveratrol treated groups and 28.06% (±6.32) in pterostilbene treated groups. The radical decrease of tumor load clearly shows the high anticancer and tumor inhibitory potential of both resveratrol and pterostilbene (Figure 3B). Tumor volumes were measured and graphed showing that the tumor size decreased by 83.5% in resveratrol treated groups and by 72% in pterostilbene treated groups (Figure 3C) as compared to control. The reduction of size for both treatment groups is significant compared to control (*p* < 0.0001) but not significantly different from each other.

### Reduction of E6 Oncoprotein Level *in vivo* by Resveratrol and Pterostilbene

**Figure 4:**
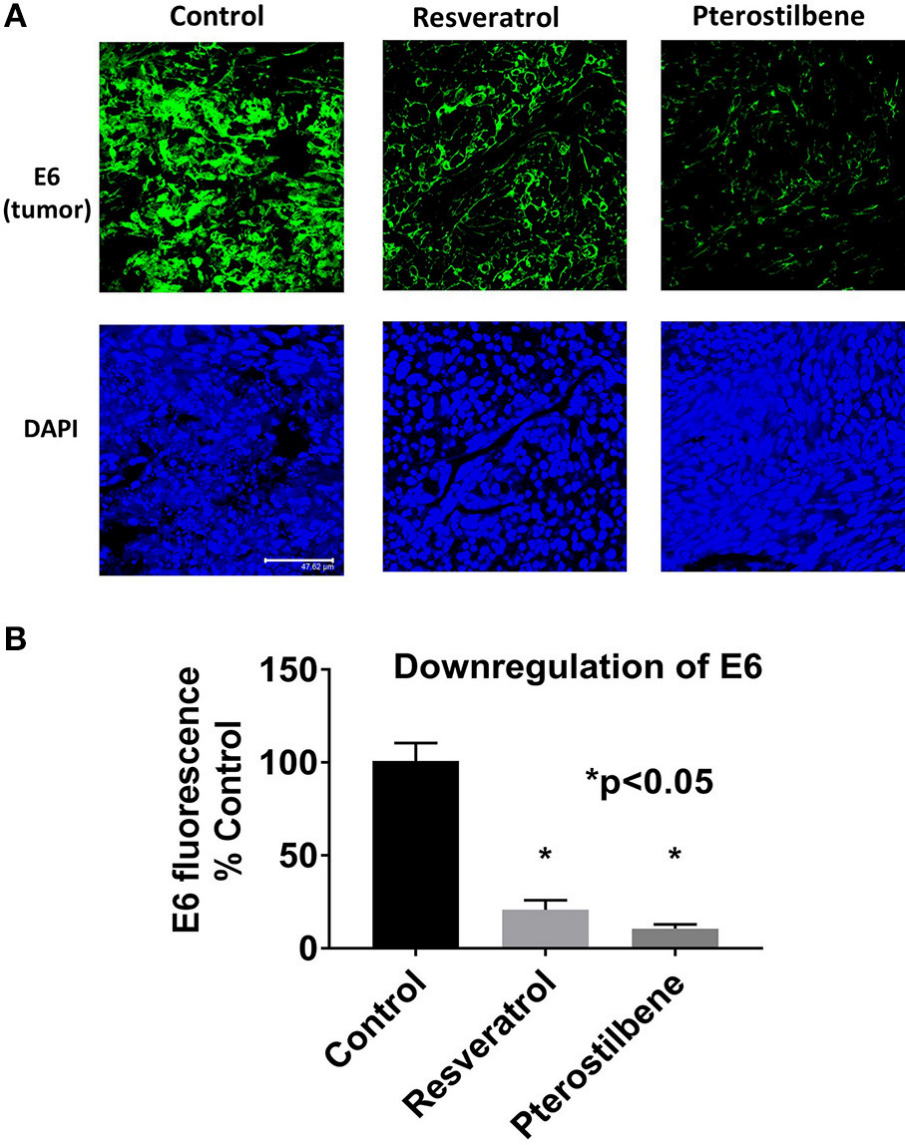
E6 expression is reduced in mouse tumors. **(A)** Tumor sections immunostained with E6 antibody shows decreased E6 protein (green) in mice treated with resveratrol or pterostilbene when compared to control untreated tumors. Sections were counterstained with DAPI (blue). Scale bar: 47.62 μm. **(B)** Graph indicates the significant reduction of E6 expression levels in resveratrol or pterostilbene treated tumors in comparison to control tumors sections (mean ± S.E.M.; ^*^p < 0.05).

To determine if E6 is downregulated in the resveratrol and pterostilbene treated tumors we used immunohistochemistry. E6 protein levels can be seen to be very high in control untreated tumor sections (Figure 4A). However, the treated groups show much lower expression of E6 indicating that both the polyphenols can target E6 for downregulation. In control tumors almost all the cells are E6 positive with high level of expression. In resveratrol and pterostilbene treatment not only are the number of E6 positive cells reduced but also the level of protein expression in these cells is lower. The fluorescence intensities of the tumor sections were compared and graphed (Figure 4B). Resveratrol has an intensity of 20.6 ± 5.1 (79% decrease vs. control) and pterostilbene has a total intensity of 10.5 ± 2.2 (89% decrease vs. control). These finding indicate that both polyphenols can significantly downregulate the expression of E6 in murine tumors compared to untreated tumors (*p* < 0.0001) and corroborate the tumor volume reduction data.

### Downregulation of PCNA Protein *in vivo* by Resveratrol and Upregulation of Cleaved Caspase 3 by Pterostilbene

**Figure 5:**
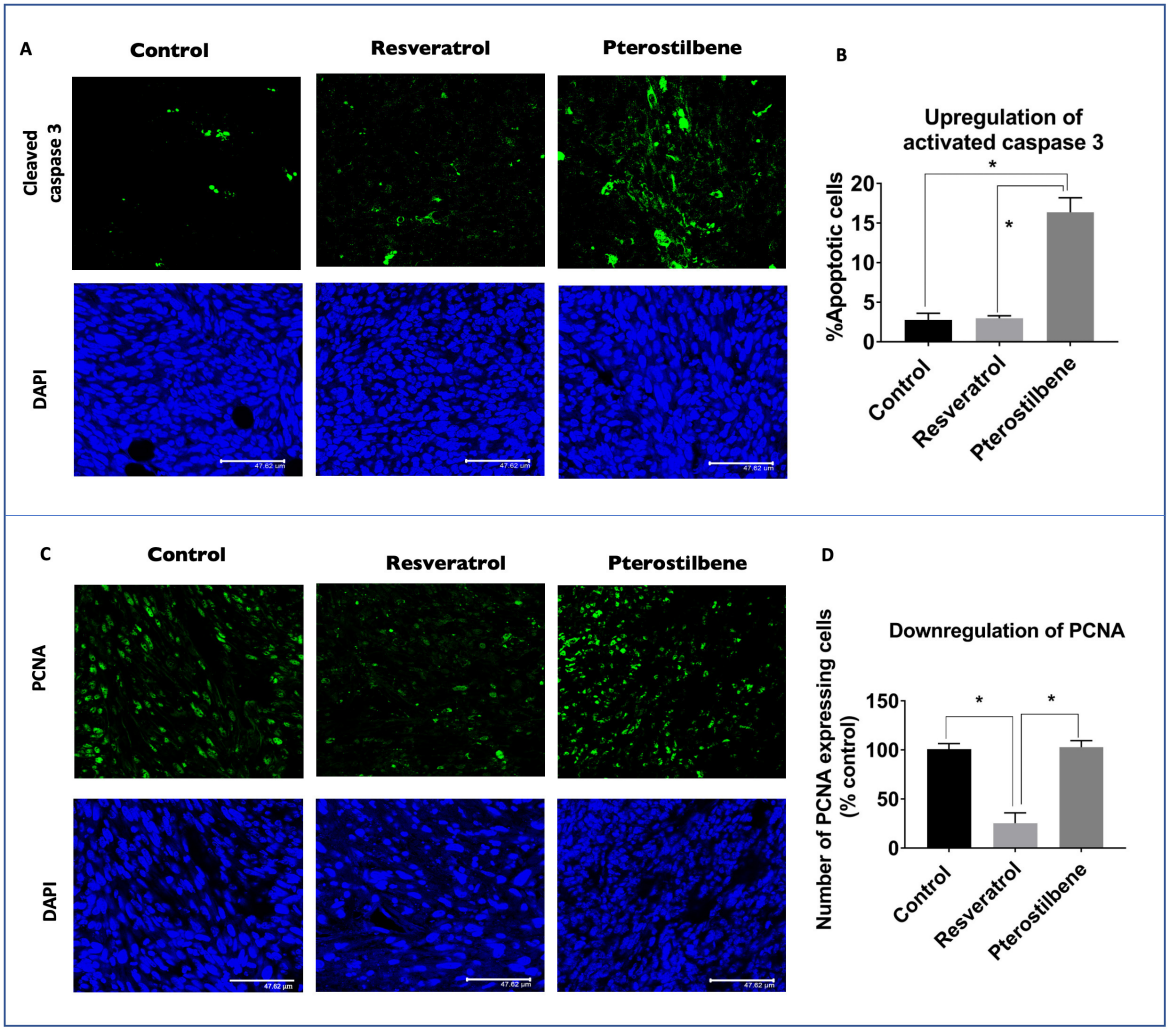
Upregulation of activated caspase 3 and downregulation of PCNA expression in mouse tumors. (**A**) Tumor sections immunostained with cleaved caspase 3 antibody shows elevated protein levels (green) in mice treated with pterostilbene when compared to control untreated tumors. Resveratrol treated tumors did not show any significant change in caspase 3 expression. Sections were counterstained with DAPI (blue). Scale bar: 47.62μm. (**B**) Graph indicates the significant increase of Cleaved caspase 3 expression levels in pterostilbene treated tumors in comparison to control tumors sections (mean ± S.E.M.; *p < 0.0001). (**C**) Tumor sections immunostained with PCNA protein (green) and counterstained with nuclear stain DAPI (blue). Resveratrol treated tumors display a significant decrease in PCNA expression compared to control sections. Pterostilbene treated tumors show similar PCNA levels as control. Scale bar: 47.62 μm. (**D**) Quantitative analysis of PCNA expression shows a significant change in resveratrol treated tumor sections (mean ± S.E.M.; *p < 0.0004; The two treatment groups show significant differences in PCNA expression (*p* < 0.0006).

To understand the underlying mechanism(s) of tumor inhibition by these two polyphenols we analyzed by immunohistochemistry the apoptotic marker, activated caspase-3 (Figures 5A,B), and the proliferation marker, PCNA (Figures 5C,D). These results indicated that tumors treated with pterostilbene have a significant amount of activated caspase-3 protein compared to resveratrol and control tumors (Figure 5A). The percentage of apoptotic cells represented graphically in Figure 5B indicates that *in vivo* resveratrol treatment does not appear to activate the process of caspase-3-mediated apoptosis whereas pterostilbene treatment significantly increased this pathway (16.34 vs. 2.72%; *p* < 0.0001). The difference in activation of caspase-3 by pterostilbene is also significantly greater than tumors treated with resveratrol (*p* < 0.0001). In contrast, resveratrol treated tumors showed a significantly lower expression of PCNA compared to untreated and pterostilbene treated tumors (Figure 5C). The percentage of cells expressing PCNA reduced by 75.5% in resveratrol treated tumors compared to control (25.26 ± 10.5 vs. 100 ± 5.6; *p* < 0.0004). Moreover, the downregulation of PCNA positive cells by resveratrol is significantly different than tumors treated with pterostilbene (*p* < 0.0006). Taken together these results show that resveratrol targets downregulation of proliferation (Figures 5C,D) whereas pterostilbene activates apoptosis in TC1 tumors, with both polyphenols providing a substantial decrease in tumor growth compared to control untreated tumors.

### Downregulation of VEGF Protein *in vivo* by Resveratrol and Pterostilbene

**Figure 6:**
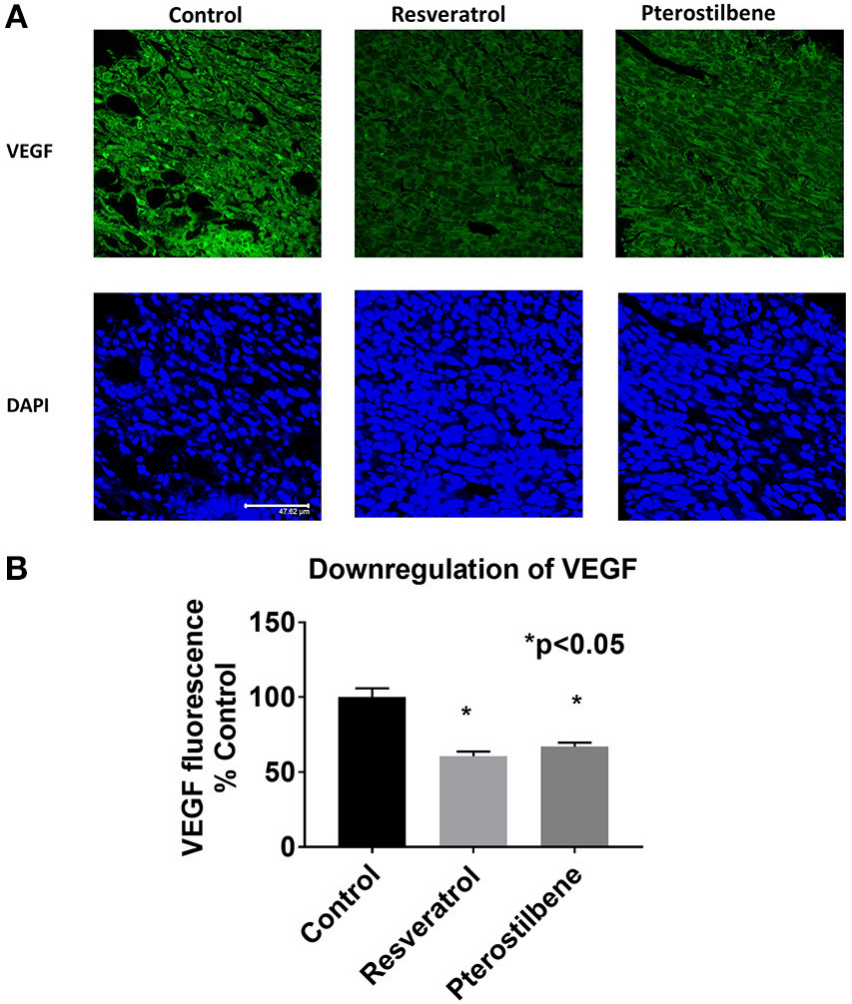
Downregulation of VEGF expression in mouse tumors. **(A)** Tumor sections immunostained with VEGF protein (green) and counterstained with nuclear stain DAPI (blue). Both resveratrol and pterostilbene treated tumors display a significant decrease in VEGF expression compared to control sections. Scale bar: 47.62 μm. **(B)** Quantitative analysis of VEGF expression shows a significant change in treated tumor sections (mean ± S.E.M.; ^*^p < 0.05).

E6 is known to upregulate VEGF expression in HPV positive tumor cells (30) therefore, we analyzed VEGF expression as a biomarker for E6 downregulation. Immunohistochemical analyses of tumor tissue showed that VEGF expression is lowered by the treatment of either resveratrol or pterostilbene (Figure 6A). Control tumor sections show a high expression of VEGF whereas treatment with either resveratrol or pterostilbene lowered the protein levels. The reduction is seen ubiquitously throughout the tumor sections in both the treated groups. The graph ascertains the level of downregulation with resveratrol showing a reduction to 60.5% (60.57 ± 3.13) and pterostilbene to 66.8% (66.88 ± 2.8) compared to control (100%) (Figure 6B). Both treatments were able to significantly downregulate the expression of VEGF protein (*p* < 0.0001).

## Discussion

In the current study we have elucidated for the first time the efficacy of stilbene compounds, resveratrol and pterostilbene using an *in vivo* murine E6 positive tumor model. We first demonstrated that *in vitro* pterostilbene shows a higher efficacy in eliminating E6-positive TC1 cells, when compared to resveratrol (Figure 1). It is important to note that both compounds show significant cytotoxicity when compared to untreated cells. Further *in vitro* analysis revealed that pterostilbene targeted significant downregulation of the HPV oncoprotein E6 (Figure 2), which drives these cells to form tumors *in vivo*. In comparison, cells treated with resveratrol exhibited less E6 compared to untreated controls, but the results were not statistically significant (Figure 2).

Our *in vivo* studies using TC1 murine model showed that both pterostilbene and resveratrol treatment cause a significant decrease of tumor size. Upon further examination we were able to demonstrate that pterostilbene causes significant apoptosis in the tumors whereas resveratrol plays a major role in arresting the growth of these tumors. Therefore, this study shows for the first time, to the best of our knowledge that resveratrol and pterostilbene can exhibit antitumor activities and reduce tumor load by two distinct mechanisms; resveratrol through cell cycle arrest (down regulation of PCNA) and pterostilbene through apoptosis (upregulation of caspase-3; Figure 5). Delving deeper into the possible targets of these polyphenols we found that the angiogenic protein VEGF is significantly reduced by both these natural compounds (Figure 6). Our study, for the first time, elucidates that both resveratrol and pterostilbene show anticancer potential *in vivo* by downregulating HPV E6.

We first confirmed the cytotoxic potential of resveratrol and pterostilbene on TC1 cells *in vitro*. Our results showed that pterostilbene is two-fold more potent than resveratrol in eliminating TC1 cells (Figure 1). This corroborated our previous findings on HeLa cells where we had seen a similar pattern of IC_50_ with pterostilbene showing superior efficacy than resveratrol (4). HPV E6 oncoprotein is known to play an essential role in cervical carcinogenesis. Both E6 and E7 protein expression is required for continuous cell proliferation and metastasis. Studies have confirmed that eliminating E6 oncoprotein pushes the cells toward cell growth arrest and apoptosis (31, 32).Several efforts are being made to use anti-E6 strategies to induce HPV cancer cell death (33, 34). Our previous study on HeLa cells showed that although both resveratrol and pterostilbene downregulate E6 expression, pterostilbene has higher inhibitory efficacy compared to resveratrol (4). In the current study we see a similar trend in E6 level suppression *in vitro*, with pterostilbene treatment showing greater suppression of E6 protein levels than resveratrol treatment (Figure 2). This increased efficacy of pterostilbene may be attributed to its structural stability. The methoxy group in pterostilbene makes it more lipophilic and more bioavailable than resveratrol (35, 36). Pharmacokinetic studies have also established the superior bioavailability of pterostilbene over resveratrol (36, 37).

Our current and previous studies using TC1 cells and HeLa cells, respectively, confirmed the anticancer potential of resveratrol and pterostilbene *in vitro*. Here our aim was to further validate these compounds as promising cervical cancer therapies and to more importantly evaluate their anticancer potential using *in vivo* model of cervical cancer. To achieve this goal, we used TC1 cells which are E6, E7, and c-Ha-Ras positive transformed mouse epithelial cells. Due to a lack of cervical cancer mouse models, TC1 cells have been widely used for creating E6-positive mouse tumors to test vaccines and other therapies (25, 27, 38). Several studies have utilized this model to understand the role the immune system and microenvironment in HPV cancers and also study immunotherapeutic strategies (28, 39, 40). Combinatorial studies using natural compounds including resveratrol has been successfully tested on TC1 tumor models (27, 28). However, there has been no study that has investigated the potential of resveratrol and pterostilbene individually on a cervical cancer model.

To evaluate the efficacy of these polyphenols *in vivo*, we established TC1 tumors in C57BL/6 mice and subjected them to intralesional treatment with 5 daily injections of 40 μl of resveratrol (1 mM) or pterostilbene (1 mM). At the end of the treatment cycle, tumors treated with either resveratrol or pterostilbene were significantly smaller than those treated with 1 × PBS alone (Figure 3). Resveratrol was able to reduce tumor size by an average of 83% while pterostilbene reduced tumor size by an average of 72%, when compared to control. The difference in tumor reduction between resveratrol and pterostilbene was not significant, which was unexpected given the potency of pterostilbene to more efficiently reduce E6 *in vitro* (Figure 2). When we assessed the E6 protein levels by using immunohistochemistry on tumor tissue we found resveratrol and pterostilbene both significantly inhibit E6 expression when compared to PBS (control) treated tumors, although resveratrol downregulated E6 to a lesser extent (Figure 4).

Several *in vivo* studies show that resveratrol has anticancer properties against breast cancer, colorectal cancer, and liver cancer in rat and mouse tumor models (23). In humans, resveratrol is well-tolerated with low toxicity and multiple clinical trials have proven the beneficial effects of this polyphenol on colorectal cancer and breast cancer to name a few (11). Pterostilbene has gained a lot of attention in recent years as a more potent bioavailable analog of resveratrol. Clinical trials also indicate the low toxicity of pterostilbene up to 250 mg/day dosage (13). Pterostilbene has shown potential in abrogating several cancers like breast and colon cancer in animal models (18). Although preclinical studies show the potency of pterostilbene on several types of cancers, there is a lack of large scale clinical studies (35).The *in vivo* efficacy of these two polyphenols on cervical cancer has also not been substantially explored. Several *in vivo* studies have explored using intra cervical treatment as a potential mode of treatment and we have tried to explore the maximum potential of resveratrol and pterostilbene at a specific dosage against HPV E6 positive cancer (41–44). Previous studies by our team have found that intravaginal application of natural compound formulation has no adverse effects and can possibly decrease HPV positive tumor load (45). Our studies bring to light the strong potential of resveratrol and pterostilbene to reduce tumor growth in a mouse cervical cancer model. The results show support toward clinical trials investigating resveratrol and pterostilbene in late stage cervical cancer, when local resection of tumor is not effective or as a possible preventative treatment to those individuals with chronic HPV cervical infection in the absence of tumor.

Suppression of apoptosis and uncontrolled proliferation are major steps essential for progression and metastasis of cancer (31). Resveratrol and pterostilbene are known to have proapoptotic, chemo preventative, and anticancer properties (46). Keeping in mind the extensive reduction of tumor growth seen *in vivo* we wanted to explore if resveratrol and pterostilbene trigger the apoptotic pathway. Several *in vitro* studies have shown that both these polyphenols can induce apoptosis in several cancer cell lines including cervical cancer (4, 24, 47, 48). Our result showed that pterostilbene but not resveratrol is able to induce considerable apoptotic cell death in TC1 tumor cells *in vivo*. The upregulation of pro-apoptotic activated caspase-3 by pterostilbene was significantly higher than untreated tumors whereas, resveratrol showed barely any change (Figure 5A). The difference in caspase-3 activation between the two treatments was statistically significant and it was surprising to us given resveratrol's potency to induce cell death in our *in vitro* studies. We then further analyzed the growth inhibitory effects of these two polyphenols in tumor tissue by performing immunohistochemistry for PCNA. PCNA is known to be highly up regulated in HPV positive cancers (49) and is known to play a major role in cancer neoplasia (50). Our results showed that resveratrol, but not pterostilbene, can drastically downregulate PCNA expression in TC1 tumors. The untreated and pterostilbene treated tumors had similar PCNA expression patterns (Figure 5B). Our findings elucidate that resveratrol and pterostilbene reduce tumor size by possibly two different mechanisms, resveratrol by downregulating growth and pterostilbene by upregulating death of tumor cells. It is known that pterostilbene is more bioavailable than resveratrol *in vivo* (36, 51). In our studies on HeLa cells we had seen that resveratrol can cause cell cycle arrest at a sub IC_50_ concentration (4). We think that possibly pterostilbene being more bioavailable is absorbed more in the tumor resulting in a higher internal concentration compared to resveratrol which being less bioavailable results in a lower intra-tumoral concentration. Based on our previous study and current results we hypothesize that resveratrol with a lower tumor concentration induces cell cycle arrest whereas pterostilbene remains at a high concentration within the cell and thus triggering apoptosis. Moreover, cells of the tumor microenvironment may respond differently to the two polyphenols to cause this similar tumor reduction (28).

HPV E6 is an oncoprotein which has several important targets that can affect cellular pathways leading to oncogenesis (52). VEGF overexpression is of supreme importance in cervical cancer biology (30). Studies have shown that HPV E6 is associated with activating the promoter for VEGF gene in cervical cancers (30). VEGF is known to induce angiogenesis, tumor cell proliferation, and plays a vital role in cervical neoplastic progression (53). Current therapies for recurrent cervical neoplasia include the usage of bevacizumab (Avastin) which is an anti-VEGF antibody (54). Unfortunately treatment with Avastin is associated with several serious side effects including hemorrhaging (54). Previous studies have shown that resveratrol can inhibit angiogenesis by targeting HIF-1α and VEGF in cervical cancers cells (54). Pterostilbene has also shown anti-angiogenic properties in skin and lung cancers (18). However, there have been no studies evaluating pterostilbene's efficacy in cervical cancer. In the current study we found that both resveratrol and pterostilbene can downregulate VEGF expression and therefore should be further evaluated as alternative or complement to current anti-angiogenic cervical cancer therapies. Both treatment groups can downregulate VEGF expression equally (Figure 6). As VEGF is an important target for therapy our findings establish the therapeutic potential of resveratrol and pterostilbene in HPV positive cancers. Further studies are required to fully understand the underlying mechanism of action of these two polyphenols and delineate the possible downstream targets triggered by E6 downregulation that elicit this tumor reduction.

Thus, our findings show the potency of resveratrol and pterostilbene on TC1 tumors by downregulating the oncoprotein E6, which drives TC1 tumor growth. The robust capability of these natural plant compounds to arrest and eliminate E6 positive tumor cells is promising for those researchers looking for alternative therapies to cervical cancer or for a better understanding of E6 oncogene activity.

## Conclusions

Cervical cancer remains prevalent in many countries where anti-HPV vaccines and preventative preneoplastic assessments are not routinely provided. Here we provide strong evidence that the natural compounds pterostilbene and resveratrol are effective in drastically shrinking a cervical cancer tumor model *in vivo* when injected directly into the tumor. Further studies, analyzing our tumor model treated with systemic injections of these compounds is the next appropriate step. Given these compounds are well-tolerated in humans, they may provide clinicians with a viable therapy in the treatment of cervical cancer, especially when current therapies are not available because of the high cost to the patient.

## Ethics Statement

This study was carried out in accordance with the NIH guidelines for animal use and approved by the Institutional Animal Care Committee (IACUC) of the College of Staten Island (CUNY) (approval # 11–008).

## Author Contributions

KC, SM, PB, and JF: conceptualization and writing—review and editing. KC, SM, and JV: data curation and methodology. KC, JV, and JF: formal analysis. PB and JF: resources. PB and JF supervision. KC and JF: writing—original draft. All authors approve the submitted manuscript.

### Conflict of Interest Statement

The authors declare that the research was conducted in the absence of any commercial or financial relationships that could be construed as a potential conflict of interest.
