# Corrigendum: Dietary polyphenols, resveratrol and pterostilbene exhibit antitumor activity on an HPV E6-positive cervical cancer model: an *in vitro* and *in vivo* analysis

**DOI:** 10.3389/fonc.2024.1483410

**Published:** 2024-10-28

**Authors:** Kaushiki Chatterjee, Sumit Mukherjee, Jonathan Vanmanen, Probal Banerjee, Jimmie E. Fata

**Affiliations:** ^1^ Doctoral Program in Biology, CUNY Graduate Center, New York, NY, United States; ^2^ Department of Biology, College of Staten Island, New York, NY, United States; ^3^ Doctoral Program in Biochemistry, CUNY Graduate Center, New York, NY, United States; ^4^ Department of Chemistry & The Center for Developmental Neuroscience, City University of New York at The College of Staten Island, New York, NY, United States

**Keywords:** HPV E6 positive cervical cancer, natural product, resveratrol, pterostilbene, PCNA, caspase-3, VEGF, *in vivo*

In the published article, there was an error in **Figure 5**, **Panel C** as published. The control panels were not accurately represented. The corrected **Figure 5** **panel C** and its caption: “(C) Tumor sections immunostained with PCNA protein (green) and counterstained with nuclear stain DAPI (blue). Resveratrol treated tumors display a significant decrease in PCNA expression compared to control sections. Pterostilbene treated tumors show similar PCNA levels as control. Scale bar: 47.62 μm.” appear below.

**Figure 5:**
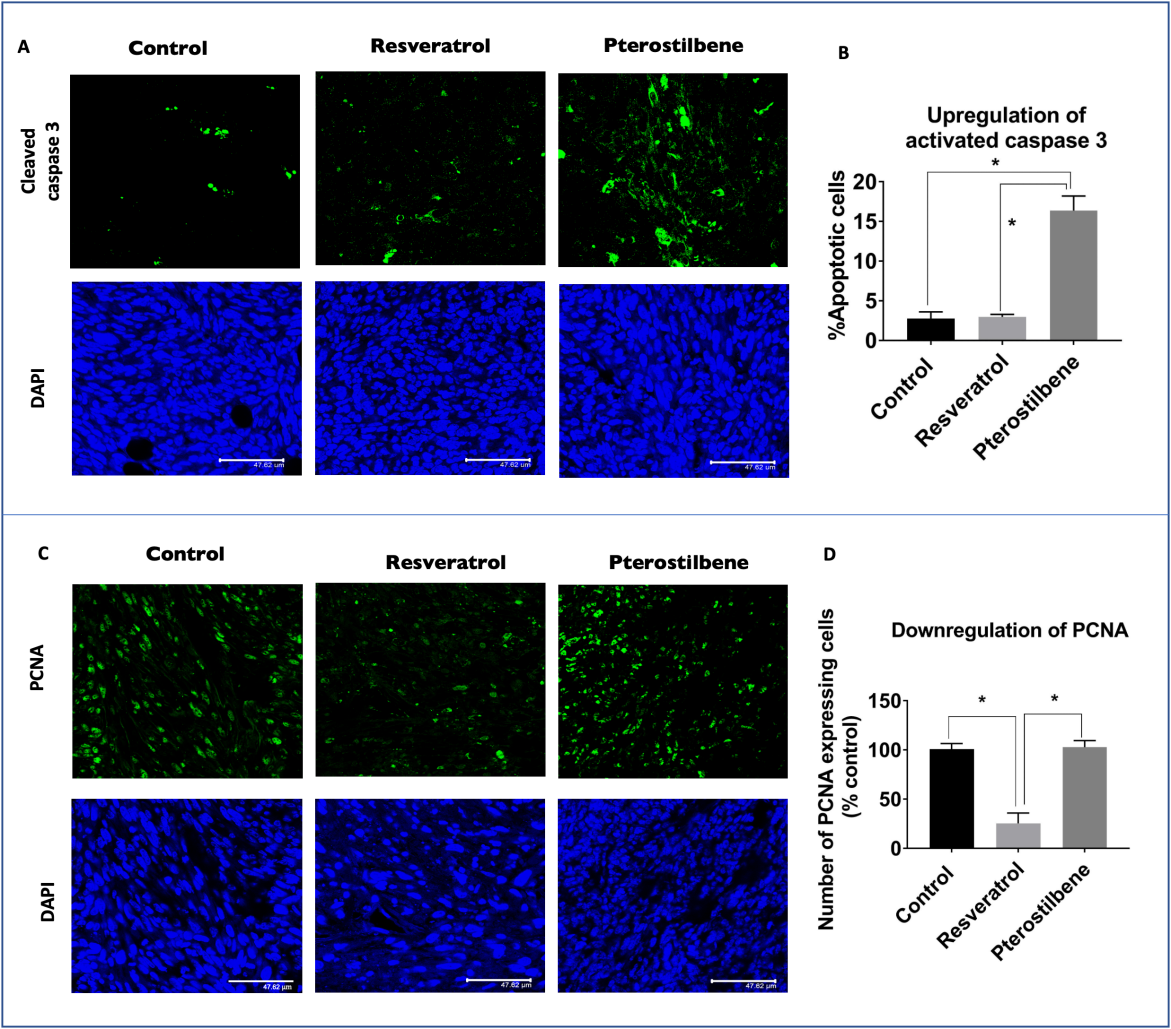
Upregulation of activated caspase 3 and downregulation of PCNA expression in mouse tumors. **(A)** Tumor sections immunostained with cleaved caspase 3 antibody shows elevated protein levels (green) in mice treated with pterostilbene when compared to control untreated tumors. Resveratrol treated tumors did not show any significant change in caspase 3 expression. Sections were counterstained with DAPI (blue). Scale bar: 47.62µm. **(B)** Graph indicates the significant increase of Cleaved caspase 3 expression levels in pterostilbene treated tumors in comparison to control tumors sections (mean ± S.E.M.; *p < 0.0001). **(C)** Tumor sections immunostained with PCNA protein (green) and counterstained with nuclear stain DAPI (blue). Resveratrol treated tumors display a significant decrease in PCNA expression compared to control sections. Pterostilbene treated tumors show similar PCNA levels as control. Scale bar: 47.62 μm. **(D)** Quantitative analysis of PCNA expression shows a significant change in resveratrol treated tumor sections (mean ± S.E.M.; *p < 0.0004; The two treatment groups show significant differences in PCNA expression (*p* < 0.0006).

The email of the corresponding author is no longer active. The correct email address of the corresponding author Kaushiki Chatterjee has been updated to kaush.mits@gmail.com.

The authors apologize for these errors and state that this does not change the scientific conclusions of the article in any way. The original article has been updated.

